# ZC3H4 regulates infiltrating monocytes, attenuating pulmonary fibrosis through IL-10

**DOI:** 10.1186/s12931-022-02134-2

**Published:** 2022-08-12

**Authors:** Yaping Liu, Xinxin Zhang, Jing Wang, Fuhuang Yang, Wei Luo, Jie Huang, Mengling Chen, Sha Wang, Caolong Li, Wei Zhang, Jie Chao

**Affiliations:** 1 Department of Physiology, Jiangsu Provincial Key Laboratory of Critical Care Medicine, Zhongda Hospital, School of Medicine, Southeast University, 87 Dingjiaqiao Rd, Nanjing, 210009 Jiangsu China; 2 Key Laboratory of Environmental Medicine Engineering, Ministry of Education, School of Public Health, Southeast University, Nanjing, 210009 Jiangsu China; 3 Key Laboratory of Development Genes and Human Disease, Southeast University, Nanjing, 210009 Jiangsu China; 4 Key Laboratory of Biomedical Functional Materials, School of Science, China Pharmaceutical University, Nanjing, 211198 Jiangsu China; 5 School of Medicine, Xizang Minzu University, Xianyang, 712082 Shanxi China

**Keywords:** Monocytes, ZC3H4, Silicosis, Autophagy, IL-10

## Abstract

Silicosis is a pulmonary fibrosis-associated disease caused by the inhalation of large amounts of free silicon dioxide (SiO_2_) that mainly manifests as early inflammation and late pulmonary fibrosis. As macrophage precursors, monocytes accumulate in the lung during early inflammation, but their role in the development of silicosis is unclear. Single-cell sequencing (cell numbers = 25,002), Western blotting, quantitative real-time PCR, ELISA and cell functional experiments were used to explore the specific effects of monocytes on fibroblasts. The CRISPR/Cas9 system was used to specifically knock down ZC3H4, a novel member of the CCCH zinc finger protein family, and was combined with pharmacological methods to explore the mechanism by which ZC3H4 affects chemokine and cytokine secretion. The results indicated that (1) SiO_2_ induced an infiltrating phenotype in monocytes; (2) infiltrating monocytes inhibited the activation, viability and migration of fibroblasts by regulating IL-10 but not IL-8; and (3) SiO_2_ downregulated IL-10 via ZC3H4-induced autophagy. This study revealed that ZC3H4 regulated the secretion function of monocytes, which, in turn, inhibited fibroblast function in early inflammation through autophagy signaling, thereby reducing pulmonary fibrosis. These findings provide a new idea for the clinical treatment of silicosis.

**Supplementary Information:**

The online version contains supplementary material available at 10.1186/s12931-022-02134-2.

## Introduction

Silicosis is a chronic occupational disease caused by long-term inhalation of free silicon dioxide (SiO_2_) [1]. Silicosis is a potentially fatal, incurable and disabling pulmonary disease that is characterized by silicosis nodule formation and pulmonary interstitial fibrosis [2]. However, as early as 1995, the International Labor Organization (ILO) and World Health Organization (WHO) proposed the "Global Pneumoconiosis International Plan", which aimed to completely eliminate pneumoconiosis by 2030. However, recently, the Lancet suggested that in recent years, the world has failed to prevent and treat pneumoconiosis [3, 4]. Moreover, the incidence and prevalence of silicosis are increasing markedly, and effective therapies are not currently available. Despite a plethora of studies that have investigated the toxicity of crystalline silica over the last several decades, the exact mechanism of silicosis currently remains elusive.

Monocytes are innate immune cells and have functions such as phagocytosis, antigen presentation and inflammation [5]. As macrophage precursor cells, monocytes accumulate in the lungs in the early stage of silicosis, helping to maintain the immune function of macrophages. In humans, monocytes can be divided into three subgroups: the inflammatory type, intermediate type and patrolling type [6]. In inflammatory diseases, patrolling monocytes also induce proinflammatory effects [7]. Bone marrow-derived monocytes accumulate at inflammatory sites along chemokine gradients and exert effects [8]. Few studies have been conducted on the function of monocytes during pulmonary fibrosis, and their functions are still controversial: some scholars believe that the release of TGF-β1 by monocytes inhibits collagen degradation and exacerbates pulmonary fibrosis [9, 10], and some scholars believe that C–C motif chemokine receptor 2 (CCR2)^+^ monocytes can inhibit lung fibrosis [11]. These findings strongly suggest that monocytes play a crucial role in silicosis. Studies [12, 13] have shown that monocytes in an inflammatory environment can affect the function of fibroblasts. This effect may be a positive way the body responds to early inflammation in silicosis by inhibiting early fibroblast activation to suppress the development of lung fibrosis in the later stage.

Zinc finger CCCH-type containing 4 protein (ZC3H4) is a novel member of the CCCH zinc finger protein family that has not been extensively studied at the preclinical or clinical levels [14]. Previous studies in our lab have shown that ZC3H4 greatly affects the progression of SiO_2_-induced endothelial-mesenchymal transition (EndoMT) via ER stress and autophagy [15]. Our study found that ZC3H4 regulates SiO_2_-induced monocyte infiltration through autophagy.

In this study, we found that ZC3H4 could regulate monocytes by reducing interleukin 10 (IL-10) release to affect fibroblast functions. These findings suggest that monocytes play an important role in the development of silicosis and that ZC3H4 can affect monocytes.

## Results

### Silica promotes an infiltrating phenotype in monocytes

**Fig. 1:**
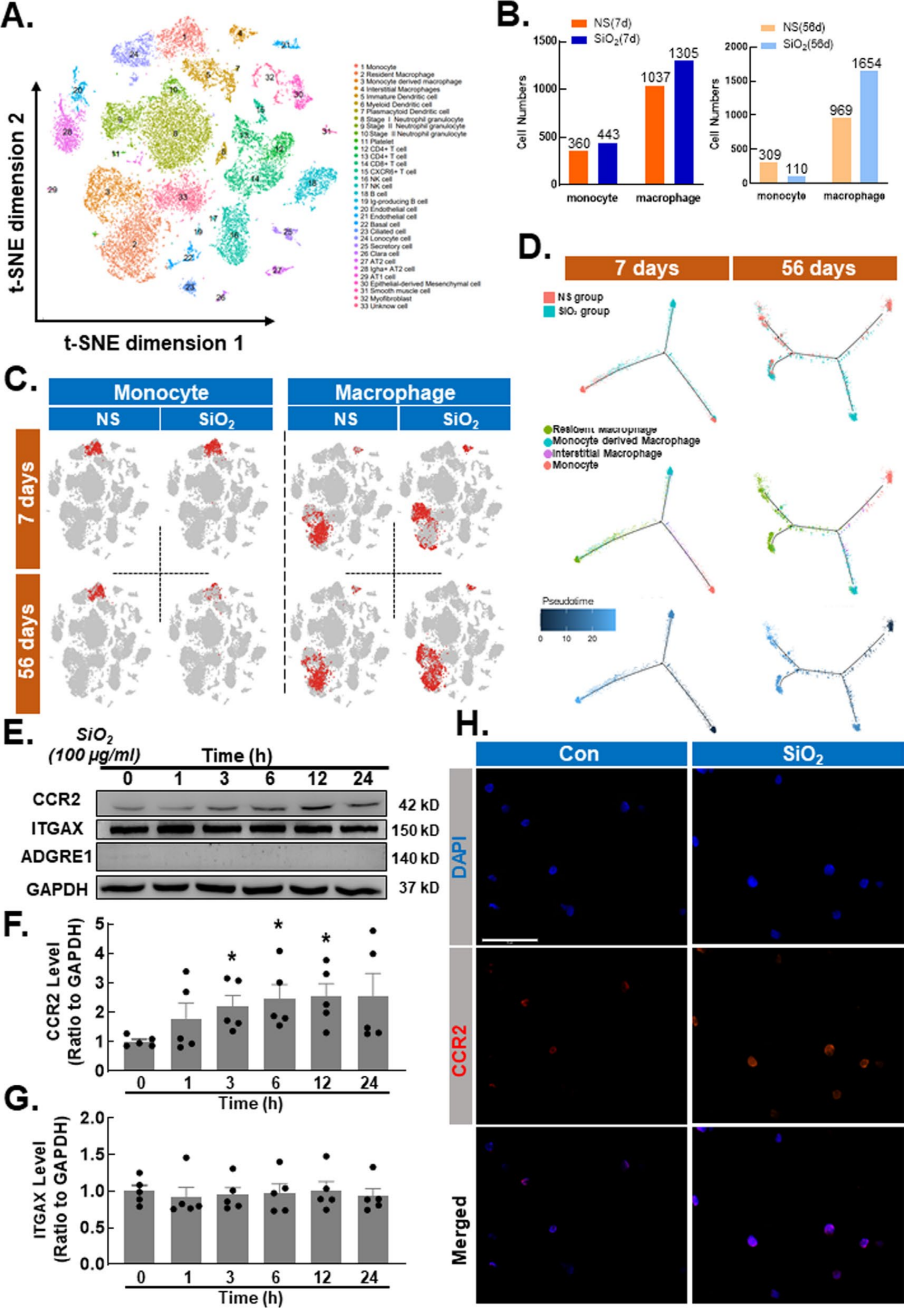
Silica promotes an infiltrating phenotype in monocytes. **A** Visualization of major classes of cells using *t*-SNE. Dots, individual cells; color, cell types. One mouse was used for each group. **B** Cell numbers and relative proportions of monocytes and macrophages are shown as pie charts at 7 d and 56 d. **C** As shown in the images, the monocyte numbers were decreased or showed no obvious difference in the SiO_2_ group compared with the NC group, while macrophage numbers increased in the SiO_2_ group. **D** Cell trajectory analysis of monocytes and macrophages and pseudotime analysis. **E** Representative Western blot showing that SiO_2_ induced CCR2 expression in a time-dependent manner in THP-1 cells. **F** Densitometric analyses of CCR2 levels from five independent experiments; *P < 0.05 compared with the 0 h group. **G** Densitometric analyses of ITGAX levels in five independent experiments. **H** Representative immunocytochemical staining images showing that SiO_2_ induced CCR2 expression in THP-1 cells. Scale bar, 100 μm

First, we used single-cell sequencing (sc-Seq) technology to examine whole lungs of mice in the 7-d saline group, 7-d SiO_2_ model group, 56-d saline group and 56-d SiO_2_ model group by digestion analysis, and all of the cell classifications in the mouse lung were obtained (Fig. 1A) by R language analysis. A 7-day mouse model of silicosis indicates acute exposure to SiO_2_, when the lungs are mainly in an inflammatory phase; in a 56-day mouse model of silicon lung, the lungs are chronically exposed to SiO_2_, mainly in the fibrosis phase [16, 17]. The number of monocytes in the 7-d SiO_2_ model group was not significantly different from that in the 7-d saline group, while the number of macrophages in the 7-d SiO_2_ model group was elevated. The number of monocytes in the 56-d SiO_2_ model group was significantly lower than that in the 56-d saline group, while the number of macrophages was elevated in the 56-d SiO_2_ model group (Fig. 1B, C). After pseudotime analysis, the increased macrophages were shown to be transdifferentiated from monocytes (Fig. 1D). Many studies have shown that alveolar macrophages play an important role in the process of fibroblast activation and the development of silicosis [18, 19]. As macrophage precursor cells, monocytes are recruited in large numbers during silicosis, but their effects are still unclear. To determine whether SiO_2_ affects the monocyte phenotype, the THP-1 cell line was exposed to SiO_2_ (100 µg/ml), and phenotypic changes were assessed. Interestingly, the immunoblotting results (Fig. 1E, F) showed that SiO_2_ upregulated CCR2 but not integrin subunit alpha X (ITGAX, CD11C, Fig. 1E, G), adhesion G protein-coupled receptor E1 (ADGRE1, F4/80, Fig. 1E) or CD163 molecule (Cd163, Additional file 1: Fig. S1A, B), suggesting that the infiltrating monocyte phenotype was increased. This finding was further confirmed by immunostaining analysis of CCR2 in THP-1 cells (Fig. 1H; Additional file 1: Fig. S1C) compared with that in the control groups, indicating that transdifferentiation from monocytes to macrophages was blocked, which was contrary to the sc-Seq results. Therefore, the role of infiltrating monocytes in the process of fibrosis is worth exploring.

### Monocytes play a negative role in fibroblast activation, migration and viability after silica treatment

**Fig. 2:**
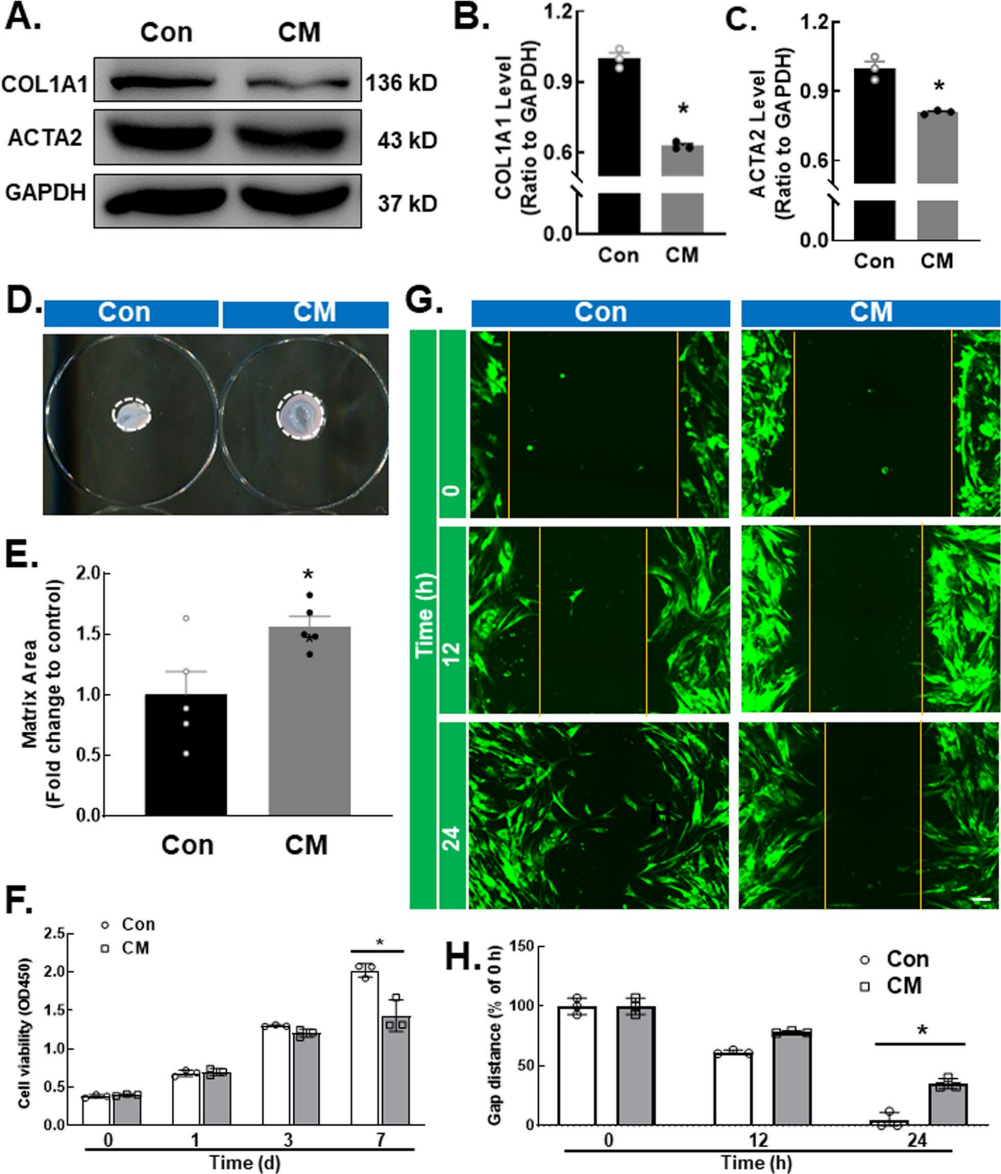
Monocytes play a negative role in fibroblast activation, migration and viability after silica treatment. **A** Representative Western blot showing the effect of CM on the upregulation of COL1A1 and ACTA2 in fibroblasts. **B** Densitometric analyses of COL1A1 levels in three independent experiments; *P < 0.05 vs. the control group. **C** Densitometric analyses of ACTA2 levels in three independent experiments; *P < 0.05 vs. the control group. **D** Representative images of the gel contraction assay of fibroblasts treated with CM. **E** Gel contraction assay results demonstrating the conditioned medium-induced decrease in fibroblasts; *P < 0.05 vs. the control group. **F** CCK-8 assay results showing that CM attenuated fibroblast viability; *P < 0.05 vs. the corresponding time point in the control group, n = 3. **G** Representative images of a scratch assay showing that the migration of fibroblasts was attenuated by CM. Scale bar, 20 μm. **H** Quantification of the scratch gap distance in three independent experiments; *P < 0.05 vs. the corresponding time point in the control group

Lung fibroblasts, which are the direct effector cells of pulmonary fibrosis, gradually transform into myofibroblasts during the progression of silicosis [20]. To determine whether infiltrating monocytes affect fibroblast activation, we treated the HPF-a cell line with conditioned medium monocytes treated with SiO_2_ (CM) or PBS (Con). The immunoblotting results demonstrated that compared with Con, CM inhibited the expression of the fibroblast activation marker proteins COL1A1 and ACTA2 (Fig. 2A–C). To determine whether infiltrating monocytes affect fibroblast viability, HPF-a cells were cultured in CM or Con. As indicated by the gel contraction assay results, the CM groups exhibited less cell viability than the Con group (Fig. 2D, E). Moreover, the CCK-8 assay results showed that CM inhibited fibroblast viability compared with that in the control group (Fig. 2F), confirming the gel contraction assay results. Considerable evidence has suggested that pulmonary fibroblast migration is one main cause of pulmonary fibrosis. We examined whether infiltrating monocytes affected fibroblast migration. The results of the scratch assay (Fig. 2G, H) showed that CM inhibited the migration of HPF-a cells. Taken together, these results suggested that monocyte exposure to SiO_2_ exerted a protective effect against fibrosis.

### Increased IL-8 expression is not a key factor that affects fibroblast activation, viability or migration

**Fig. 3:**
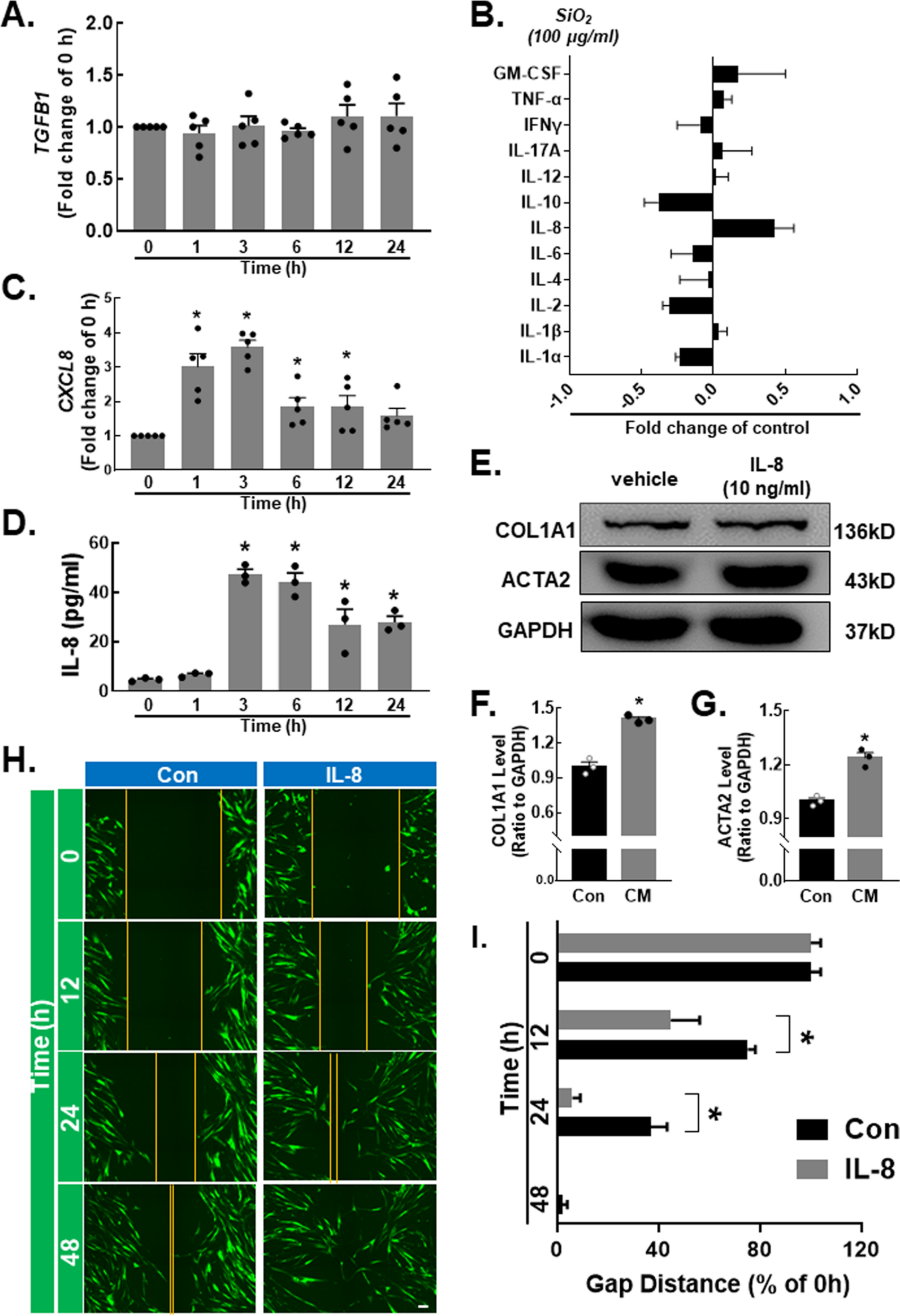
Increased IL-8 expression is not a key factor that affects fibroblast activation, viability and migration. **A** RT‒qPCR analysis showing that SiO_2_ stimulation had no effect on *TGFB1* expression. **B** SiO_2_ induced the expression of 12 inflammatory factors in THP-1 cells. **C** RT‒qPCR analysis showing that *cxcl8* expression was increased in THP-1 cells in response to SiO_2_ stimulation (n = 5); *P < 0.05 vs. the 0 h group. **D** ELISA analysis showing that IL-8 protein expression was increased in THP-1 cells in response to SiO_2_ stimulation (n = 3); *P < 0.05 vs. the 0 h group. **E** Representative Western blot showing the effect of IL-8 on the upregulation of COL1A1 and ACTA2 in fibroblasts. **F** Densitometric analyses of COL1A1 protein expression levels in three independent experiments; *P < 0.05 vs. the control group. **G** Densitometric analyses of ACTA2 protein expression levels in three independent experiments; *P < 0.05 vs. the control group. **H** Representative images from the scratch assay showing that the migration of fibroblasts was increased by IL-8. Scale bar, 20 μm. **I** Quantification of the scratch gap distance in three independent experiments; *P < 0.05 vs. the corresponding time point in the control group

To further investigate which cytokines influence the functions of fibroblasts, we examined several factors. First, we measured the classic cytokine TGF-β1 [21, 22] and found that the mRNA level was not changed after SiO_2_ stimulation (Fig. 3A). Then, we measured 12 inflammation-related cytokines by ELISA and found an increase in IL-8 expression (Fig. 3B). To confirm whether IL-8 was increased by SiO_2_, we measured its mRNA and protein expression. The results showed that IL-8 mRNA expression (Fig. 3C) and IL-8 protein levels increased (Fig. 3D) in the THP-1 cell line after SiO_2_ stimulation. To identify whether IL-8 was the main factor in CM that affected fibroblasts, we added IL-8 to normal medium. Interestingly, both ACTA2 and COL1A1 were significantly increased after IL-8 treatment (Fig. 3E–G). In addition, IL-8 also promoted fibroblast viability (Figure S2A). The 2D scratch assay was used to evaluate migration, and IL-8 promoted cell migration, as expected (Fig. 3H, I).

### Decreased IL-10 release is a key factor that affects fibroblast activation, viability and migration

**Fig. 4:**
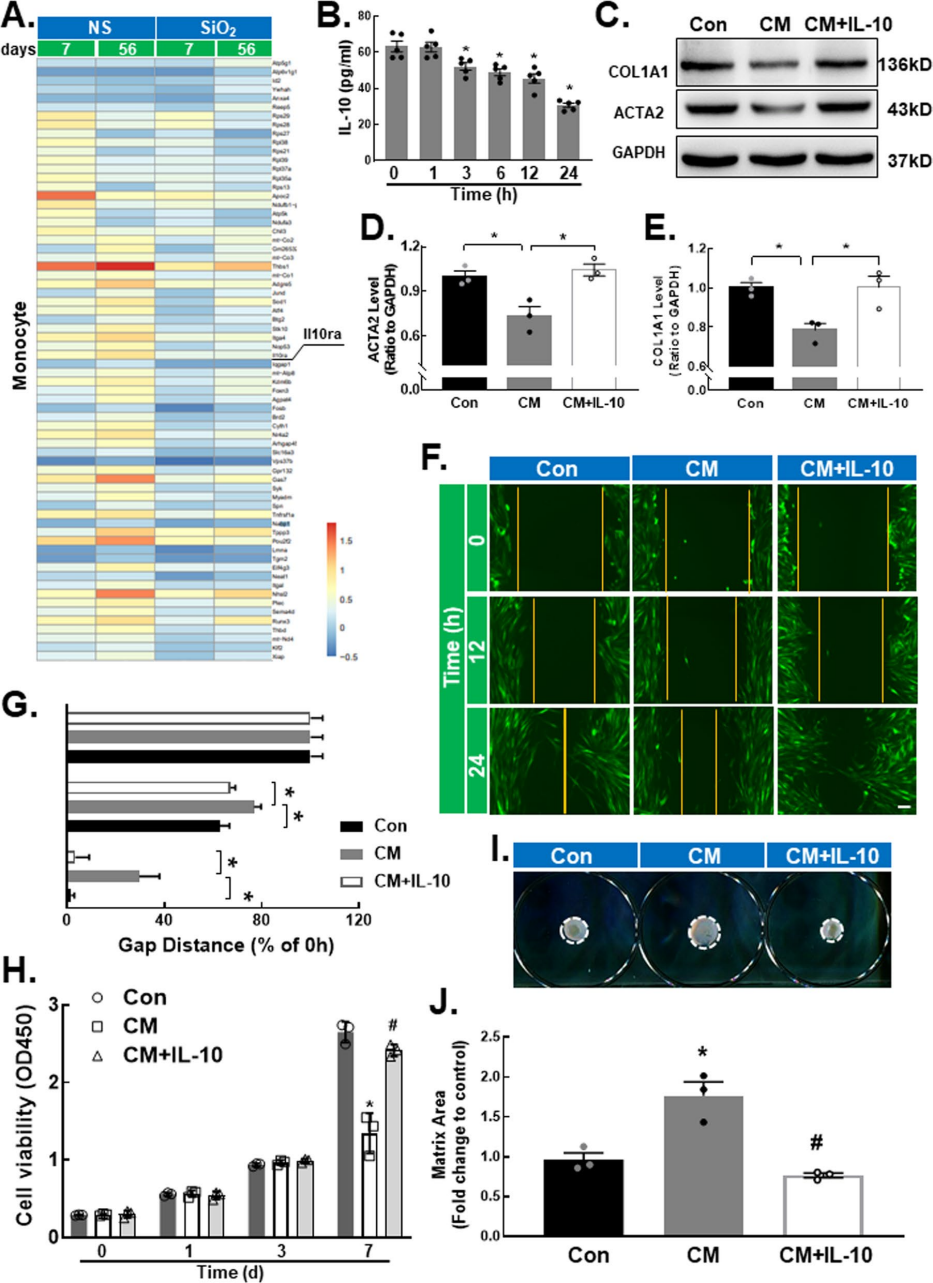
Decreased IL-10 release is a key factor that affects fibroblast activation, viability and migration. **A** IL10ra in the NS group and the SiO_2_ group is shown in the heatmap of the monocyte cluster. **B** ELISA analysis showing that SiO_2_ decreased IL-10 protein expression in THP-1 cells (n = 5); *P < 0.05 vs. the 0 h group. **C** Representative Western blot showing the effect of CM and IL-10 on the specific upregulation of COL1A1 and ACTA2 in fibroblasts. **D** Densitometric analyses of ACTA2 levels in three independent experiments; *P < 0.05 vs. the control group, *P < 0.05 vs. the CM group. **E** Densitometric analyses of COL1A1 levels in three independent experiments; *P < 0.05 vs. the control group, *P < 0.05 vs. the CM group. **F** Representative images from the scratch assay showing that the migration of fibroblasts was increased by CM and IL-10. Scale bar, 20 μm. **G** Quantification of the scratch gap distance in three independent experiments; *P < 0.05 vs. the corresponding time point in the control group. **H** CCK-8 assay results showing that fibroblast viability was increased by CM and IL-10; *P < 0.05 vs. the corresponding time point in the control group, #*P* < 0.05 vs. the corresponding time point in the CM group, n = 3. **I** Representative images of gel contraction assays showing fibroblasts treated with CM and IL-10. **J** Gel contraction assay results demonstrating the decrease in fibroblast viability induced by CM and IL-10; *P < 0.05 vs. the control group, #*P* < 0.05 vs. the CM group, n = 3

After examining the effect of IL-8, we found that it was inconsistent with the effect of CM; thus, we reviewed the previous data and shifted our focus to the decreased fibrosis factor IL-10 [19, 23, 24] (Fig. 3B). We analyzed the differentially expressed genes in monocyte populations by single-cell sequencing and found that the expression of IL-10 receptors was decreased in the model group (Fig. 4A). To confirm that the change in IL-10 was induced by SiO_2_, we measured IL-10 protein levels and found a time-dependent decrease in response to SiO_2_ stimulation (Fig. 4B). To further determine the role of IL-10, we added IL-10 to CM. As expected, the fibroblast activation marker proteins ACTA2 and COL1A1 were both restored after the addition of IL-10 to CM (Fig. 4C–E). In addition, IL-10 promoted the migration of HPF-a cells compared with those treated with CM (Fig. 4F, G). In addition, the CCK-8 assay (Fig. 4H) and gel contraction assay (Fig. 4I, J) results indicated that IL-10 could abrogate the negative effects of CM on HPF-a cell viability.

### Autophagy is involved in the silica-induced reduction in IL-10 release by monocytes

**Fig. 5:**
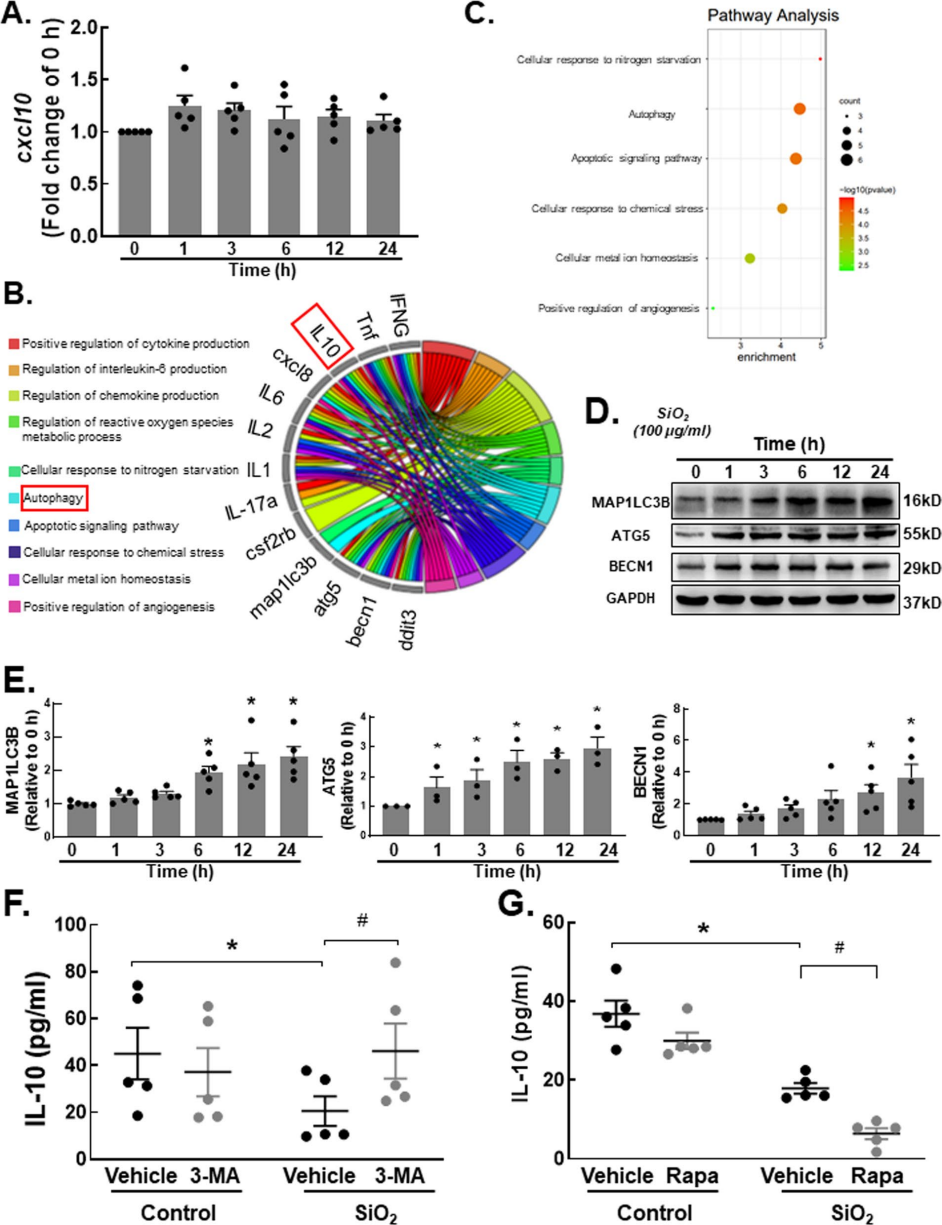
Autophagy is involved in the silica-induced reduction in IL-10 release by monocytes. **A** RT‒qPCR analysis showing that SiO_2_ stimulation had no effect on *cxcl10* expression. **B**, **C** As shown in the string map and bubble map, IL-10 plays an important role in autophagy signaling pathways. **D** Representative Western blot showing the effect of SiO_2_ on the upregulation of MAP1LC3B, ATG5 and BECN1 in THP-1 cells. **E** Densitometric analyses of MAP1LC3B levels in five independent experiments; *P < 0.05 vs. the 0 h group. Densitometric analyses of ATG5 levels in five independent experiments; *P < 0.05 vs. the 0 h group. Densitometric analyses of BECN1 levels in five independent experiments; *P < 0.05 vs. the 0 h group. **F** ELISA analysis showing that the SiO_2_-induced reduction in IL-10 protein release by THP-1 cells was reversed by the autophagy blocker 3-MA (n = 5), *P < 0.05 vs. the corresponding group in the control group. #*P* < 0.05 vs. the con group and the SiO_2_ group. **G** ELISA analysis showing that the SiO_2_-induced reduction in IL-10 protein release by THP-1 cells was promoted by the autophagy agonist rapamycin (n = 5), *P < 0.05 vs. the corresponding group in the control group. #*P* < 0.05 vs. the con group and the SiO_2_ group

Based on these results, we showed that the cytokine that affected fibroblast function was IL-10, but how silica regulated the release of IL-10 by THP-1 cells was unclear. To determine what affected the expression of IL-10, we first measured the mRNA level of IL-10. Unexpectedly, the results demonstrated that IL-10 mRNA levels did not show significant changes (Fig. 5A; Additional file 1: Fig. S3A). Based on a literature review, we initially thought that a posttranslational modification might regulate IL-10 expression. First, we were concerned that endoplasmic reticulum (ER) stress could control the release of cytokines to a certain extent [25, 26]. To verify our hypothesis, we used Western blotting to analyze ER stress markers, but no significant difference was found in the expression of the marker proteins HSPA5 and DDIT3 (Additional file 1: Fig. S3B–D). Then, we moved to the autophagy pathway, which is highly associated with cytokine release [27, 28]. We obtained a string map (Fig. 5B) and bubble map (Fig. 5C) through biological information technology and found that IL-10 played an important role in autophagy signaling pathways. To explore whether autophagy regulated the expression of IL-10, we first measured the expression of autophagy markers. The immunoblotting results showed upregulation of the markers MAP1LC3B, BECN1 and ATG5 (Fig. 5D, E). Furthermore, after using the autophagy inhibitor 3-MA to block autophagy, the SiO_2_-induced decrease in IL-10 release was significantly inhibited, and the IL-10 protein level was restored (Fig. 5F). Moreover, after treatment with the autophagy agonist rapamycin, IL-10 showed a further decline in the SiO_2_ treatment group (Fig. 5G). Notably, the level of IL-10 decreased in the vehicle group after drug administration, which may be due to the nonspecific effect of the drug on cell viability (Additional file 1: Fig. S4A, B).

### ZC3H4 regulates the silica-induced release of IL-10 by monocytes

**Fig. 6:**
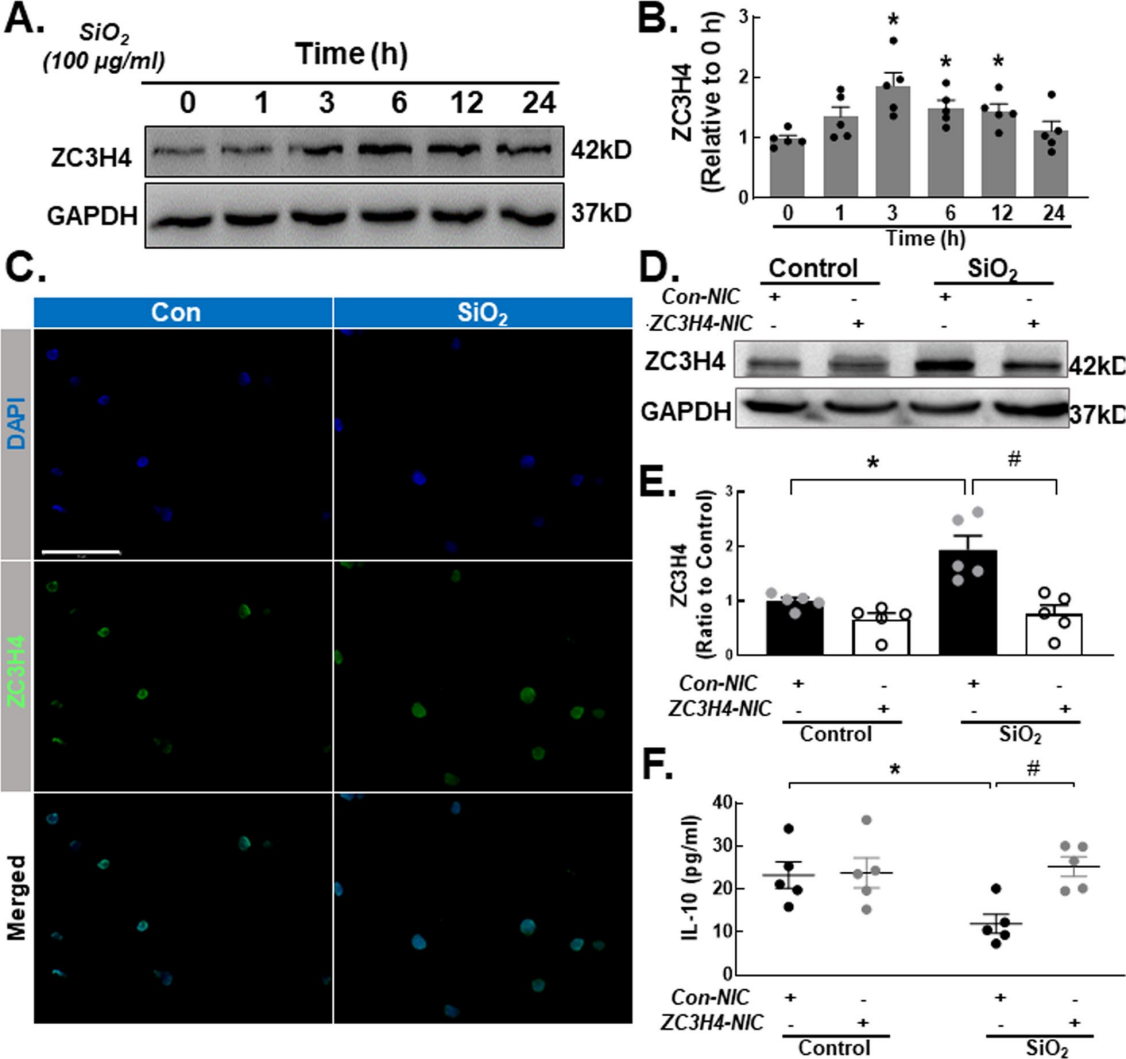
ZC3H4 is involved in regulating the silica-induced release of IL-10 by monocytes. **A** Representative Western blot showing that SiO_2_ induced ZC3H4 expression in a time-dependent manner in THP-1 cells. **B** Densitometric analyses of ZC3H4 protein expression levels in five independent experiments; *P < 0.05 vs. the 0 h group. **C** Representative immunocytochemical staining images showing that SiO_2_ induced ZC3H4 expression in THP-1 cells. Scale bar, 100 μm. **D** Representative Western blot showing that ZC3H4 protein was knocked down in THP-1 cells after plasmid transfection. **E** Densitometric analyses of ZC3H4 protein expression levels in five independent experiments; *P < 0.05 vs. the corresponding group in the control group. #*P* < 0.05 vs. the Con-NIC^+^ ZC3H4-NIC^−^ group and the SiO_2_ group. **F** ELISA analysis showing that knocking down the ZC3H4 protein increased IL-10 protein release by THP-1 cells. *P < 0.05 vs. the corresponding group and the control group. #*P* < 0.05 vs. the Con-NIC^+^ ZC3H4-NIC^−^ group and the SiO_2_ group

Previous studies in our laboratory have shown that the zinc finger proteins MCPIP1 (ZC3H12A) and ZC3H4 play important roles in the process of fibrosis caused by the inflammatory response in macrophages [19, 29]. Whether the zinc finger protein ZC3H4 is involved in the effect of monocytes on fibrosis is unclear. To identify whether ZC3H4 is involved in monocytes, we first measured ZC3H4 protein levels. The immunoblotting results showed that ZC3H4 was significantly increased after SiO_2_ treatment (Fig. 6A, B). Immunostaining also confirmed this effect (Fig. 6C and Figure S5A). CRISPR/Cas9 technology (Fig. 6D, E) was used to knock down the ZC3H4 protein (NIC). Moreover, ZC3H4-NIC upregulated the IL-10 expression level to that of the control group in the presence of SiO_2_ (Fig. 6F).

### ZC3H4 regulates IL-10 release through autophagic processes

**Fig. 7:**
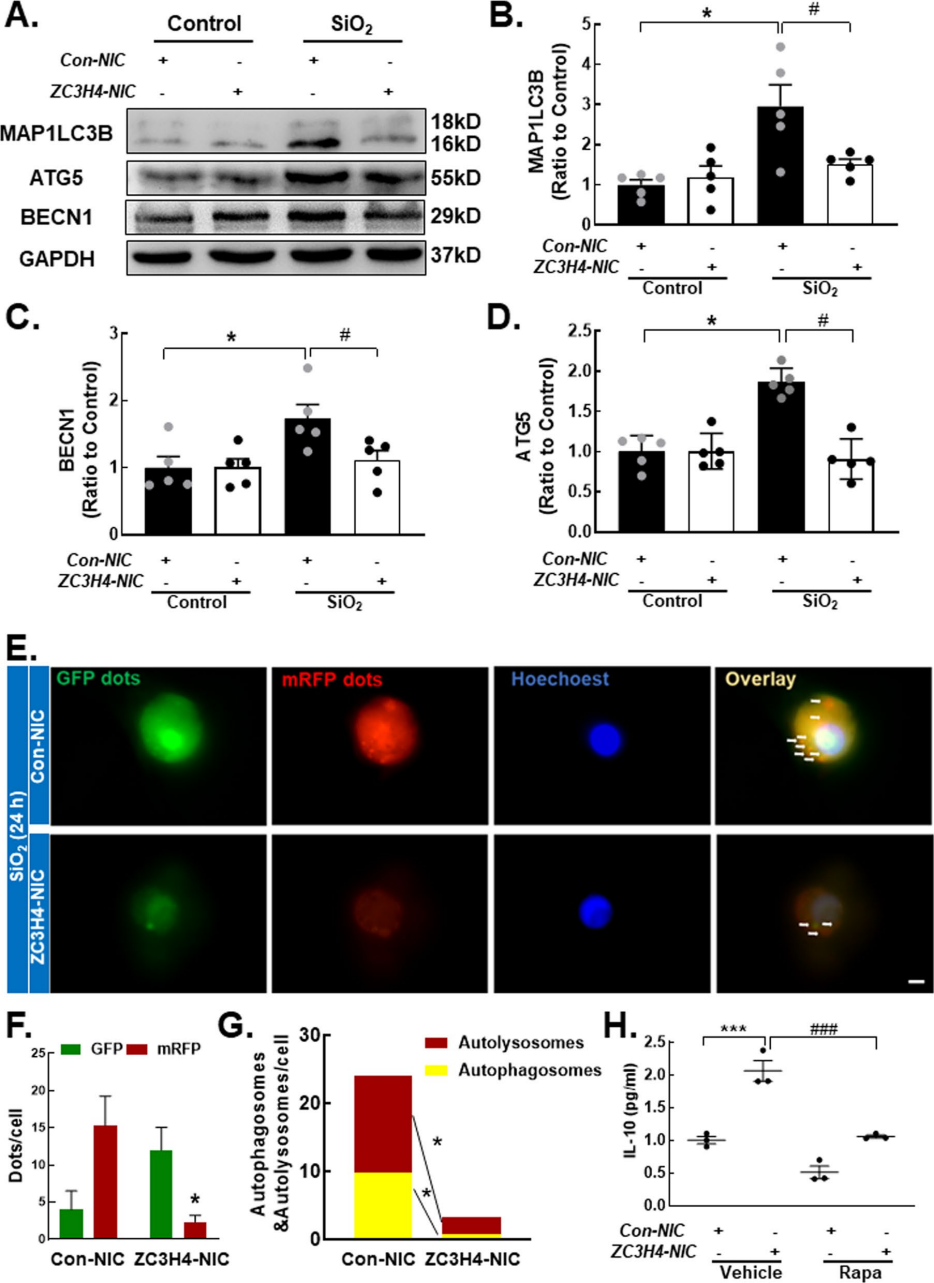
ZC3H4 regulates IL-10 release through autophagic processes. **A** Representative Western blot showing that knocking down ZC3H4 downregulated MAP1LC3B, ATG5 and BECN1 expression levels in THP-1 cells treated with SiO_2_. **B** Densitometric analyses of MAP1LC3B protein expression levels in five independent experiments; *P < 0.05 vs. the corresponding group and the control group. #*P* < 0.05 vs. the Con-NIC^+^ ZC3H4-NIC^−^ group and the SiO_2_ group. **C** Densitometric analysis of BECN1 protein expression levels in five independent experiments; *P < 0.05 vs. the corresponding group and the control group. #*P* < 0.05 vs. the Con-NIC^+^ ZC3H4-NIC^−^ group and the SiO_2_ group. **D** Densitometric analysis of ATG5 protein expression levels in five independent experiments; *P < 0.05 vs. the corresponding group and the control group. #*P* < 0.05 vs. the Con-NIC + ZC3H4-NIC- group and the SiO_2_ group. **E** Representative images of the fluorescence map showing that autophagy was attenuated in THP-1 cells stimulated with SiO_2_ and ZC3H4 protein knockdown. Scale bar, 80 μm. **F** Quantification of autophagy levels in THP-1 cells treated with SiO_2_ after ZC3H4 protein knockdown; *P < 0.05 vs. the corresponding color in the CON-NIC group. **G** Quantification of autolysosomes and autophagosomes in THP-1 cells treated with SiO_2_ after ZC3H4 protein knockdown; *P < 0.05 vs. the corresponding color in the CON-NIC group. **H** ELISA analysis showing that the autophagy agonist rapamycin decreased IL-10 protein release in THP-1 cells treated with SiO_2_ after ZC3H4 protein knockdown. ***P < 0.001 vs. the Con-NIC^+^ Rapa^−^ group. ###*P* < 0.001 vs. the NIC^+^ Rapa^−^ group

Based on these findings, the results showed that ZC3H4 and autophagy could both regulate the expression of IL-10. According to the literature, associations exist between various zinc finger proteins and autophagy. To verify the relationship between ZC3H4 and autophagy, we knocked down ZC3H4 and observed changes in autophagy marker proteins. The results suggested that the autophagy-related proteins MAP1LC3B, BECN1, and ATG5 were all decreased in the SiO_2_ group (Fig. 7A–C). To further validate this result, THP-1 cells were transduced with dual fluorescent mRFP-GFP-MAP1LC3 adenovirus to detect autophagy by monitoring autophagosome formation in real time with fluorescence microscopy. mRFP was used to label and track LC3, whereas GFP fluorescence is sensitive to acidic conditions; thus, GFP fluorescence will be quenched when a lysosome and an autophagosome form an autolysosome. Controls that were not SiO_2_-stimulated had almost no obvious autophagic flux (Additional file 1: Fig. S5D, F). SiO_2_ significantly induced autophagic flux, and this effect of SiO_2_ was attenuated by knocking down ZC3H4 (Fig. 7D, F). Moreover, after knocking down ZC3H4, rapamycin reduced the expression of IL-10 under SiO_2_ stimulation compared with the effect of no rapamycin.

## Discussion

Alveolar macrophages are a particular group of macrophages within lung tissue that respond to particles that are inhaled through the pulmonary bronchial airway via intricate interactions with other cells, such as fibroblasts and epithelial cells [30]. These macrophages function as effector cells by secreting and releasing factors that attract and regulate other cells, resulting in continuous increases in mesenchymal components [31]. According to the different states of macrophages in the inflammatory response, they can be divided into [32] classically activated M1 types and selectively activated M2 macrophages. M1 macrophages have a strong proinflammatory ability and bactericidal effect [33], while M2 macrophages exhibit strong anti-inflammatory activity [34]. Many efforts have been made to prevent fibrosis in the context of silicosis. However, no effective therapies or drugs are currently available to prevent or minimize the progression of SiO_2_-induced inflammation.

As macrophage precursor cells, monocytes are innate immune cells that have functions such as phagocytosis, antigen presentation and inflammation [5]. Bone marrow-derived monocytes accumulate in the inflammatory site along chemokine gradients, thereby promoting inflammation. The cell group classifications obtained by single-cell sequencing showed that monocytes differentiated into macrophages through processes that were affected by silicone. However, when we stimulated monocytes directly with SiO_2_, monocytes were converted to the CCR2^+^ inflammatory phenotype and did not differentiate into macrophages. In a mouse silicosis model, the SiO_2_ suspension drips from the trachea into the lungs, stimulating macrophages in the lungs, which secrete inflammatory factors that recruit monocytes from the bloodstream into the lungs. At this time, the monocytes in the lungs are affected not only by SiO_2_ but also by other factors in the entire lung microenvironment. This may make the in vitro effect of SiO_2_ on monocytes different from the in vivo effect of single-cell sequencing. However, this result led us to focus on CCR2^+^ monocytes. To date, extensive evidence indicates that CCR2^+^ cells can promote fibrosis in the lungs. One study suggested that CCR2^+^ monocytic myeloid-derived suppressor cells (M-MDSCs) inhibited collagen degradation and promoted lung fibrosis by producing TGF-β1 [10]. However, our results indicated that infiltrating monocytes inhibited fibroblast activation, viability and migration. Notably, many studies have suggested that macrophages are the main effector cells that cause pulmonary fibrosis, and blocking or reducing macrophages could attenuate pulmonary fibrosis [35–38]. Based on previous studies and experimental results, we hypothesized that regulating monocyte differentiation may be an effective strategy for inhibiting inflammation and fibrosis. Interfering with monocyte differentiation could, on the one hand, suppress the inflammatory cascade; on the other hand, undifferentiated monocytes could directly play a beneficial role by inhibiting inflammation and fibrosis. If the above hypotheses are confirmed, they will provide new ideas for the treatment of silicosis.

IL-6 is a multifunctional cytokine that regulates the immune response, hematopoiesis, acute response and inflammation and can be produced by and affect a variety of cell types [39]. IL-6 is elevated in the inflammatory response, which is associated with complex immunomodulatory networks in the body. While silica stimulates monocytes in vitro and the expression of IL-6 decreases, we suspect two reasons for this: first, the secretion of IL-6 in the inflammatory process of silicosis mainly comprises macrophages, and second, the internally constructed inflammatory environment is different from the complex and changeable external environment, which may also be another important reason for the reduction in IL-6 secretion. IL-2 was discovered in 1976 as a T-cell growth factor1. IL-2 can affect the differentiation of T helper 9 (T_H_9) cells [40] and T_reg_ cells [41], which in turn inhibits the differentiation of T_H_17 cells [42, 43]. One hypothesis is that [44] IL-2 can control inflammation by inhibiting T_H_17 differentiation. We speculate that IL-2 has an anti-inflammatory effect in an inflammatory environment.

IL-10 is widely expressed in a variety of immune cells and is known as an anti-inflammatory cytokine that can inhibit the expression of a variety of inflammatory factors [45–47]. However, studies have suggested that the long-term release of large amounts of IL-10 exacerbates the progression of silicosis [23, 24, 48]. In our study, we found that the release of IL-10 by infiltrating monocytes was decreased, which partly restrained fibroblast functions. Accordingly, inhibiting fibroblast activation in the early inflammation stage may result in weakening lung fibrosis in later stages.

Autophagy, an evolutionarily conserved and catabolically driven cytoprotective process, is associated with many physiological processes, including immune cell responses to endogenous and exogenous pathogenic stimuli [49–52]. Our recent study suggested that autophagy plays a major role in determining cellular fate in silicosis [19, 20]. Furthermore, autophagy can regulate the release of various cytokines, including IL-1α, IL-1β, and IL-18 [28, 53, 54]. In our study, we found that autophagy could affect the expression of IL-10 via ZC3H4, which is a new type of zinc finger protein whose structure is unclear. In previous studies, ZC3H4 promoted macrophage activation and therefore affected downstream fibroblasts [29], and ZC3H4 was also involved in epithelial-mesenchymal transition (EMT) [14]. In this study, we found that ZC3H4 could regulate the autophagy pathway to inhibit IL-10 release in monocytes, indicating the complicated and key role of ZC3H4 in pulmonary fibrosis.

**Fig. 8:**
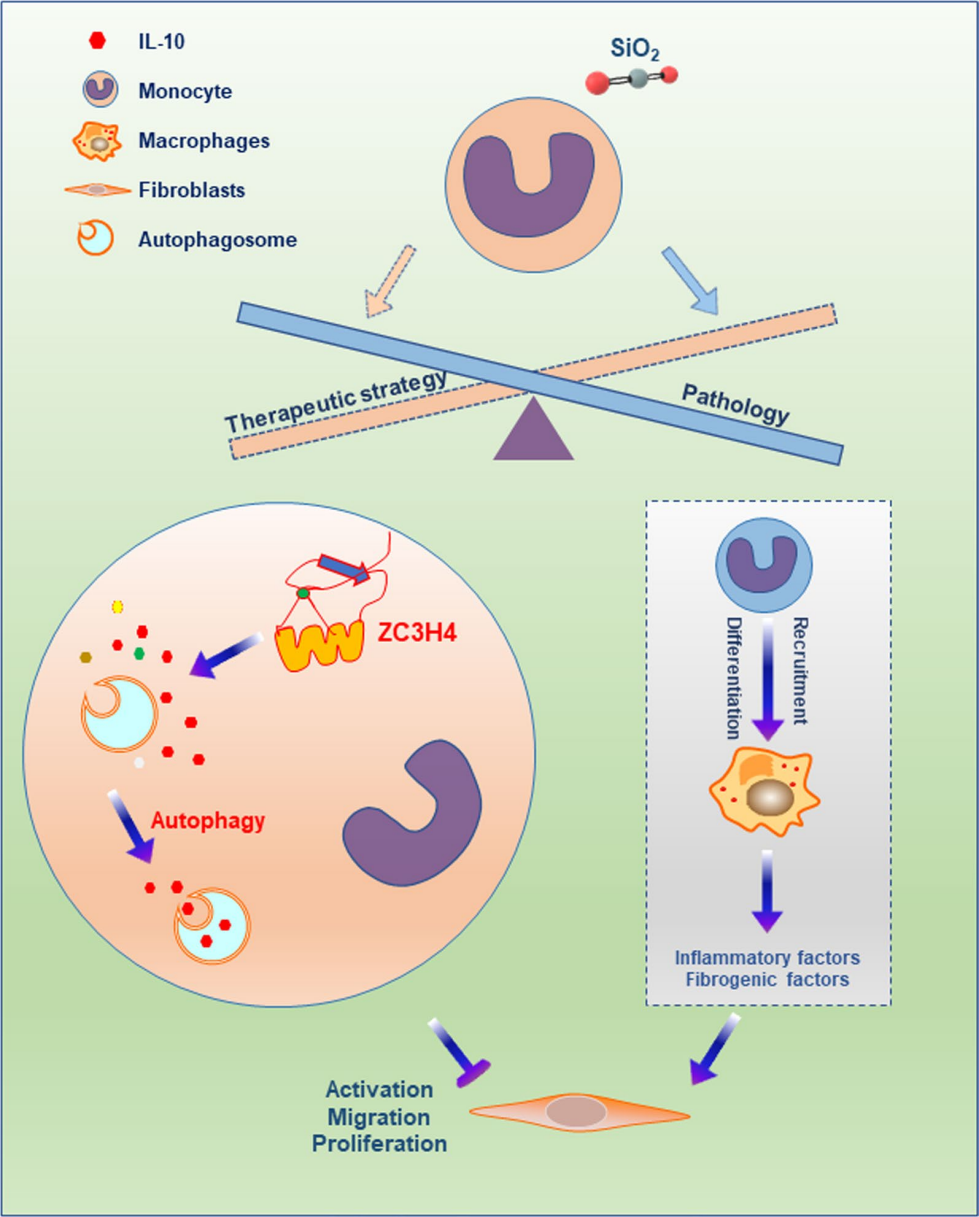
Schematic diagram showing that infiltrating monocytes can inhibit fibroblast activation, viability and migration and then inhibit lung fibrosis in the context of silicosis. ZC3H4 is a crucial protein associated with silicosis that can regulate IL-10 release by controlling autophagy in monocytes. Consequently, the regulation of monocyte differentiation might be a potential therapeutic strategy for inhibiting inflammation and fibrosis and would bring new opportunities for the treatment of silicosis

In summary, our study revealed that infiltrating monocytes could inhibit fibroblast activation, viability and migration and inhibit lung fibrosis in the context of silicosis. Additionally, ZC3H4 is a crucial protein associated with silicosis that can regulate IL-10 release by controlling autophagy in monocytes. Consequently, the regulation of monocyte differentiation might be a potential therapeutic strategy for inhibiting inflammation and fibrosis and would bring new opportunities for the treatment of silicosis (Fig. 8).

## Materials and methods

### Reagents

SiO_2_, which has a diameter of approximately 2–5 μm, was purchased from Sigma (S5631). The silica was sterilized overnight (200 °C for 16 h) [55] and then dissolved in sterile normal saline (NS) at a concentration of 5 mg/ml. The dose of SiO_2_ used in vivo and in vitro was based on previous studies [29]. Antibodies against α-SMA (14395-1-AP, rabbit), CCR2 (16153-1-AP, rabbit), BECN (11306-1-AP, rabbit), ATG5 (60061-1-lg, mouse) and LC3 (14600-1-AP, rabbit) were obtained from ProteinTech, Inc. Antibodies against collagen I (BS1530, rabbit) and GAPDH (MB001, mouse) were obtained from BioWorld, Inc.

### Animals

C57BL/6 mice (6–8 weeks old) were obtained from Dr. Tao Cheng at Nanjing Medical University Laboratories (Nanjing, China). All animals were male and housed (4 per cage) in a temperature-controlled room (25 °C, 50% relative humidity) with a 12-h light/dark cycle. All animal procedures were performed in strict accordance with ARRIVE guidelines, and animal protocols were approved by the Institutional Animal Care and Use Committee of Southeast University.

### Single-cell sequencing

1. Sample collection
  For the model group, mice with significant lesions on CT were included. Lesions were removed from the lungs of mice representing the NS-7d, SiO_2_-7d, NS-56d, and SiO_2_-56d groups and were used for single-cell sequencing. Each lung was removed in 2 min and quickly washed in precooled PBS 3 times.
2. Single-Cell RNA Sequencing.

### Cell capture and cDNA synthesis

Using a single-cell ‘5’ Library and Gel Bead Kit (10 × Genomics, 1000169) and Chromium Single-Cell G Chip Kit (10 × Genomics, 1000120), the cell suspension (300–600 living cells per microliter determined by Countstar) was loaded onto a Chromium single-cell controller (10 × Genomics) to generate single-cell gel beads in the emulsion according to the manufacturer’s protocol. In short, single cells were suspended in PBS containing 0.04% BSA. Approximately 20,000 cells were added to each channel, and the target cell recovered was estimated to be approximately 10,000 cells. Captured cells were lysed, and the released RNA was barcoded through reverse transcription in individual GEMs. Reverse transcription was performed on a S1000TM Touch Thermal Cycler (Bio Rad) at 53 °C for 45 min, followed by 85 °C for 5 min and holding at 4 °C. cDNA was generated and then amplified, and quality was assessed using an Agilent 4200 (performed by CapitalBio Technology, Beijing).

### Single-cell RNA-Seq library preparation

According to the manufacturer’s instructions, single-cell RNA-seq libraries were constructed using the Single Cell 5’ Library and Gel Bead Kit, Single Cell V(D)J Enrichment Kit, Human T-Cell (1000005) and Single Cell V(D)J Enrichment Kit. The libraries were finally sequenced using an Illumina NovaSeq6000 sequencer with a sequencing depth of at least 100,000 reads per cell with a paired-end 150 bp (PE150) reading strategy (performed by CapitalBio Technology, Beijing).

## Data preprocessing

### Cell ranger pipeline

Cell Ranger software (v.4.0.0) was obtained from the 10 × Genomics website https://support.10xgenomics.com/single-cell-gene-expression/software/downloads/latest. Pipeline coupled with mouse reference version mm10 was used. Alignment, filtering, barcode counting, and UMI counting were performed with the Cell Ranger count module to generate a feature-barcode matrix and determine clusters. Dimensionality reduction was performed using PCA, and the first ten principal components were used to generate clusters by the K-means algorithm and graph-based algorithm.

### DEGs identification and enrichment analysis

Differentially expressed genes were analyzed using sc-Seq with negative binomial models to estimate the false discovery rate (FDR). For each cluster, genes with adjusted log_2_-fold change > 3 and P < 0.001 were considered significantly upregulated. GO enrichment and KEGG enrichment of cluster markers were performed using the R package clusterProfiler, using the top significantly upregulated genes of each cluster. The results were visualized using the R package.

### Cell type annotation

Cell type was annotated by Cell Marker.

#### Cell culture

The THP-1 cell line was purchased from ATCC^®^, routinely maintained in RPMI (10% FBS, 1% penicillin/streptomycin) and incubated at 37 °C and 5% CO_2_. HPF-a cells were purchased from ATCC^®^, routinely maintained in DMEM (10% FBS, 1% penicillin/streptomycin) and incubated at 37 °C and 5% CO_2_.

#### Western blot analysis

Cells were collected in polyethylene tubes and briefly washed with ice-cold phosphate-buffered saline (PBS) twice before being lysed. The protein concentrations of the lysates were measured with a bicinchoninic acid (BCA) kit (Beyotime, China), and 30 μg of total protein was resolved via SDS‒PAGE. Then, the proteins were transferred onto a polyvinylidene difluoride (PVDF) membrane. The membrane was blocked with 5% nonfat dry milk in Tris-buffered saline with Tween-20 (TBST) for 1 h and then incubated overnight (16 h) with primary antibodies against Col1A1, α-SMA, CHOP, BIP, BECN, ATG5 and LC3B (1:1000). After being washed with TBST, the membrane was incubated for 1 h with secondary antibodies conjugated to horseradish peroxidase. Protein bands were visualized using a chemiluminescence detection system. All Western blots are representative of three or more independent experiments. The protein bands were quantified using ImageJ 1.52v software.

#### Cell viability assay

Cell viability was measured using CCK-8 assays. Briefly, the cells were seeded in 96-well plates at a density of 1 × 10^5^ cells/well (for THP-1 cells) or 5 × 10^4^ cells/well (for HPF-a cells). The cells were treated with conditioned media for 24 h (for HPF-a cells) or with 3-MA or rapamycin for 24 h (for THP-1 cells). The cells were then exposed to CCK-8 solution (10 μL), and the plates were incubated for an additional 30 min to 4 h. The absorption values were measured at 450 nm.

### Immunocytochemistry

Cells were fixed with 4% paraformaldehyde in PBS on ice for 2 h. The fixed samples were permeabilized for 30 min at room temperature (RT) in PBS containing 0.3% Triton X-100 (PBST) and then blocked with 10% normal goat serum (NGS; Life Technologies) in PBST at RT for 2 h. The blocked samples were incubated for 4 h on ice with primary antibodies diluted in PBST plus 10% NGS. The samples were then washed three times with PBS and incubated with donkey anti-rabbit (conjugated to Alexa Fluor^®^ 488) and donkey anti-mouse (conjugated to Alexa Fluor^®^ 576) secondary antibodies for 2 h at RT. After being washed three times in PBS, the samples were mounted with Prolong^®^ Gold antifade reagent containing DAPI, and the slides were examined using a fluorescence microscope.

#### CRISPR/Cas9 technology

THP-1 cells were quickly transfected with CRISPR/Cas9 plasmids according to the manufacturer’s protocol (Santa Cruz^®^) to delete ZC3H4 and observe its downstream effects. Western blotting was used to determine the transfection efficiency. In brief, 24-well plates were used for cell seeding (2 × 10^5^ cells/well), and the cells reached 40–80% density. The medium was changed to 200 μl fresh antibiotic-free growth medium, and solutions A and B were added as follows. For solution A, transfection reagent (1 μl) was poured into plasmid transfection medium (9 μl), and for solution B, plasmid DNA (1 μl) was poured into plasmid transfection medium (9 μl). After 5 min, solution A was poured dropwise directly into solution B; the sample was then immediately vortexed and incubated at room temperature for > 20 min. The mixed solution was added dropwise to 200 μl of the medium in the 24-well plate, and the contents of the well were mixed by swirling the plate gently. The medium was added or replaced when necessary 12 h after transfection. THP-1 cells were incubated for an additional 24–72 h to conduct further experiments.

#### Gel contraction assay

Fibroblast-populated collagen matrix (FPCM) contraction was determined using the floating matrix contraction assay as described previously in [56] with minor modifications. Briefly, the matrices were polymerized, covered with DMEM containing 5% FBS, released from the culture well using a sterile spatula, and incubated at 37 °C. At different time points after the matrices were released, they were fixed in 4% paraformaldehyde in PBS at 4 °C overnight, and images were obtained using a desktop flatbed scanner. The matrix area was measured using ImageJ software, and the data are presented as the ratio of the released matrix area to the attached matrix area.

#### Detection of autophagic flux

THP-1 cells were seeded in 6-well plates and transfected with mRFP-green fluorescent protein (GFP)-LC3 adenoviral vectors according to the manufacturer’s instructions (HanbioInc, Shanghai, CN, USA). Successfully transfected cells expressed LC3 protein tagged with RFP and GFP. GFP is acid-sensitive, and the green fluorescence is quenched in the acidic environment of a lysosome. However, in contrast, RFP is relatively stable within lysosomes. Therefore, the numbers of GFP and RFP puncta were examined and quantified by confocal microscopy. The red and yellow (i.e., a combination of red and green) spots indicate autophagosomes and autolysosomes, respectively [57].

#### Scratch assay

HPF-a cells were treated with conditioned media, IL-8 or IL-10 for 48 h in 24-well plates. To assess fibroblast motility, a scratch assay was performed as previously described [58].

#### Real-time PCR

Total RNA was isolated from cells and subjected to reverse transcription using a Prime Script RT master mix kit (TaKaRa, RR036). Real-time PCR was performed by a StepOne™ Real-Time PCR System (Life Technologies, 4376357, Singapore) using primers for human IL-10 (forward primer: 5′-GTGATGCCCCAAGCTGAGA-3′; reverse primer: 5′-CACGGCCTTGCTCTTGTTT-3′) and human IL-8 (forward primer: 5′-CTGATTTCTGCAGCTCTGTG-3′; reverse primer: 5′-GGGTGGAAAGGTTTGGAGTATG-3′).

#### ELISA

Twelve inflammatory cytokines were analyzed by Human Inflammatory Cytokines Multi-Analyte ELISArray™ Kits (QIAGEN, MEH-004A). Human IL-10 and human IL-8 ELISA kits were purchased from JinYiBai^®^ (Nanjing, China). All cytokines were measured according to the manufacturer’s protocol.

#### Statistical analysis

The data were analyzed using GraphPad Prism 5.0 (GraphPad Software, Inc.). Unpaired numerical data were analyzed by unpaired Student’s t tests (2 groups) or ANOVA (2 groups). The level of significance was set at 0.05; values of *P* < 0.05 indicated statistical significance.

## Supplementary Information

**Additional file 1: Fig. S1. **Silica promotes the mononuclear cell infiltration phenotype (refer to Fig. 1). **Fig. S2**. Increased IL-8 expression is not a key factor that affects fibroblast activation, viability or migration (refer to Fig. 3). **Fig. S3**. Autophagy is involved in the silica-induced reduction in IL-10 release by monocytes (refer to Fig. 5). **Fig. S4**. The autophagy inhibitor 3-MA and the autophagy agonist rapamycin affect cell viability (refer to Fig. 5). **Fig. S5**. ZC3H4 regulates IL-10 release through autophagic processes (refer to Figs. 6, 7).

## Data Availability

All of the relevant raw data and materials are freely available to any investigator upon request.

## Abbreviations

CCR2: C-C motif chemokine receptor 2
sc-Seq: Single-cell sequencing
ITGAX: Integrin subunit alpha X, CD11c
ADGRE1: Adhesion G protein-coupled receptor E1, F4/80
ER stress: Endoplasmic reticulum stress
MCPIP1: Monocyte chemotactic protein-1-induced protein, ZC3H12A
EMT: Epithelial-mesenchymal transition
SiO_2_: Silicon dioxide
ECM: Extracellular matrix
CM: Conditioned medium
HPF-a: Human pulmonary fibroblast-adult
DMEM: Dulbecco's modified Eagle's medium
NS: Normal saline
FBS: Fetal bovine serum
NGS: Normal goat serum
BALF: Bronchoalveolar lavage fluid
