# Supplementary material for: ZC3H4 regulates infiltrating monocytes, attenuating pulmonary fibrosis through IL-10
Source: Respir Res. 2022 Aug 12;23:204. doi: 10.1186/s12931-022-02134-2 (PMC9375388; doi:10.1186/s12931-022-02134-2)
Supplement: Supplementary file 1 — Additional file 1: Fig. S1. Silica promotes the mononuclear cell infiltration phenotype (refer to Fig. 1). Fig. S2. Increased IL-8 expression is not a key factor that affects fibroblast activation, viability or migration (refer to Fig. 3). Fig. S3. Autophagy is involved in the silica-induced reduction in IL-10 release by monocytes (refer to Fig. 5). Fig. S4. The autophagy inhibitor 3-MA and the autophagy agonist rapamycin affect cell viability (refer to Fig. 5). Fig. S5. ZC3H4 regulates IL-10 release through autophagic processes (refer to Figs. 6, 7). [file 12931_2022_2134_MOESM1_ESM.docx]

**ZC3H4 regulates infiltrating monocytes, attenuating pulmonary fibrosis through IL-10**

Yaping Liu^1,2,3*^, Xinxin Zhang^1*^, Jing Wang^1,2*^, Fuhuang Yang^1,*#^, Wei Luo^1*^, Jie Huang^1,2^, Mengling Chen^1^, Sha Wang^1^, Caolong Li^4^*, Wei Zhang^1^*, Jie Chao^1,2,3,5^*

Supplementary Figure S1 2

Supplementary Figure S2 3

Supplementary Figure S3 4

Supplementary Figure S4 5

Supplementary Figure S5 6

**Supplementary Figure 1.**

**
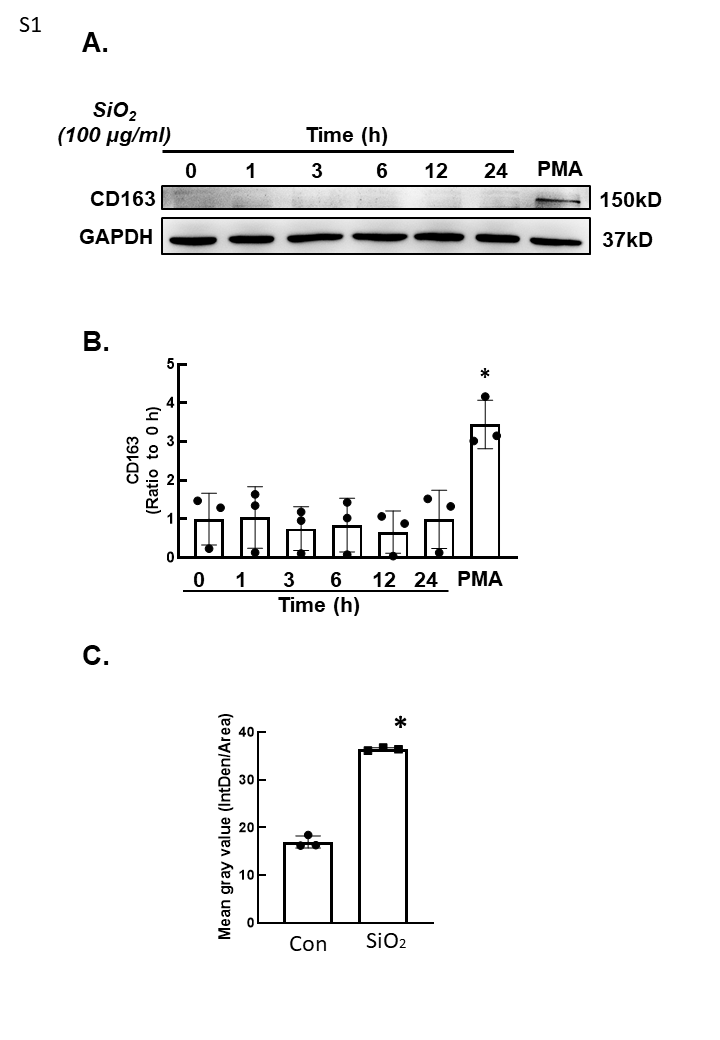
**

**Figure S1. Silica promotes the mononuclear cell infiltration phenotype (refer to Figure 1).** (A) Representative Western blot showing that SiO_2_-treated THP-1 monocytes do not express CD163, and PMA-treated THP-1 macrophages express CD163. (B) Densitometric analyses of CD163 levels from three independent experiments; **P* < 0.05 compared with the 0 h group. (C) Immunofluorescence staining quantification of CCR2 protein expression, **P* < 0.05 compared with the Con group.

**Supplementary Figure 2.**

**
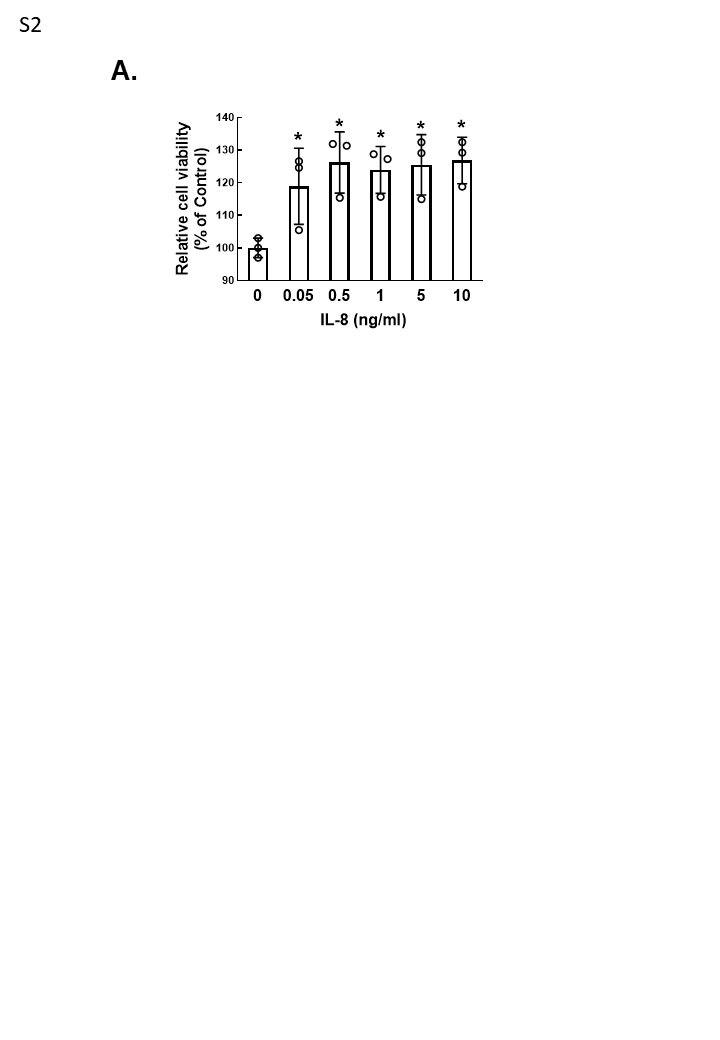
**

**Figure S2. Increased IL-8 expression is not a key factor that affects fibroblast activation, viability or migration (refer to Figure 3).** CCK-8 assay results showing that fibroblast viability was increased by IL-8; **P* < 0.05 vs. the 0 ng/ml group, n=3.

**Supplementary Figure 3.**

**
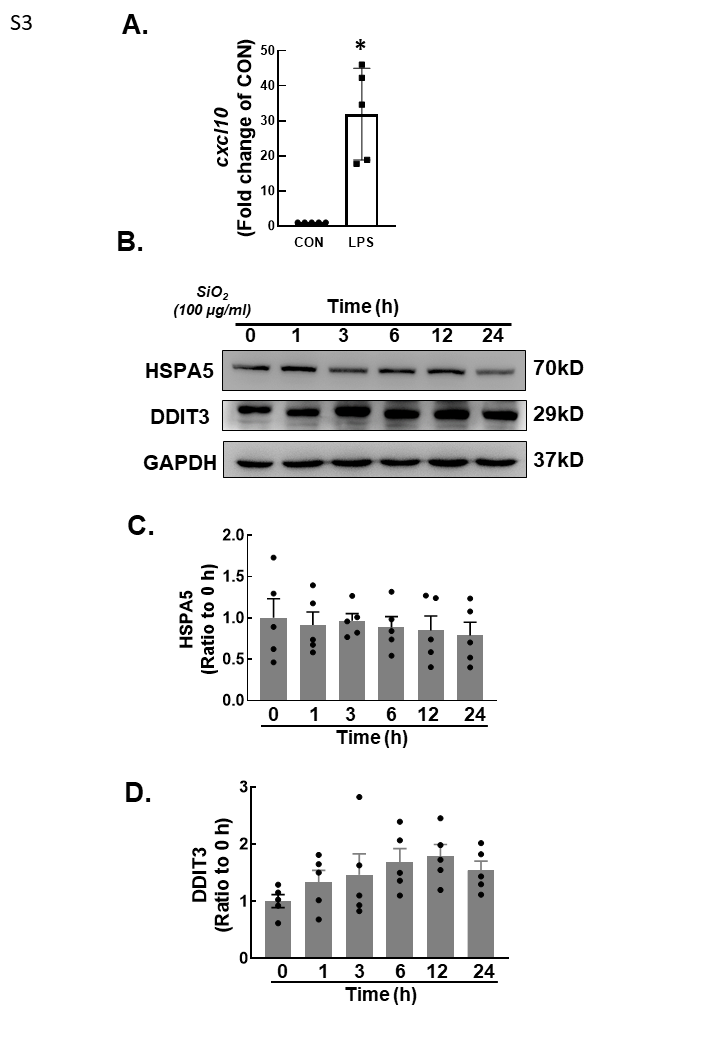

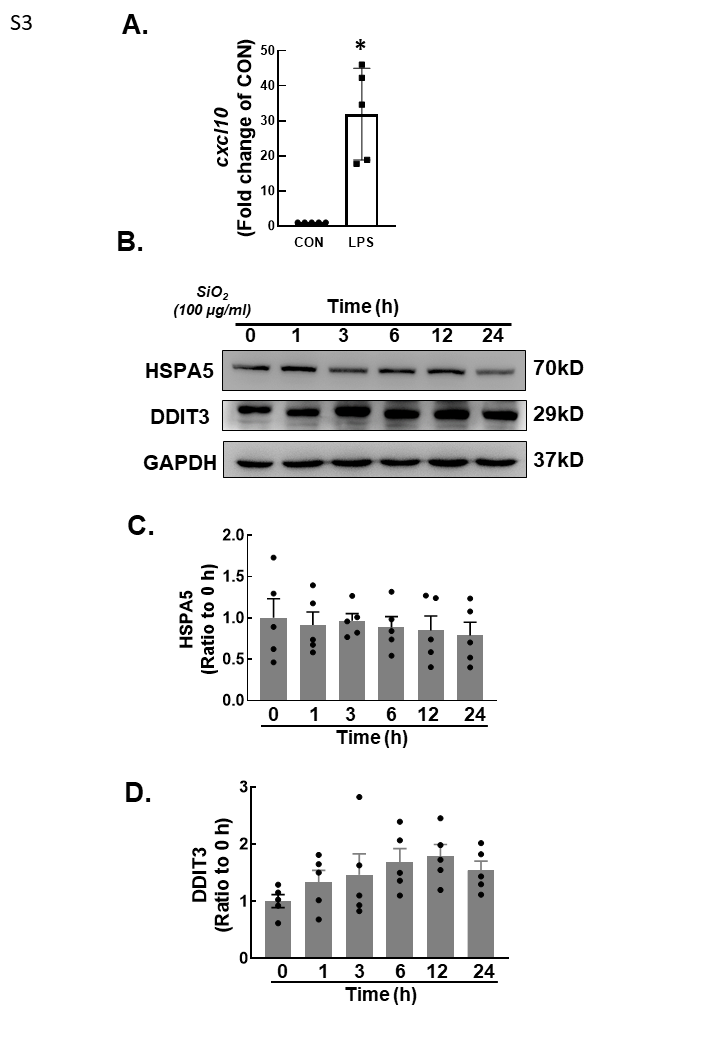
**

**Figure S3. Autophagy is involved in the silica-induced reduction in IL-10 release by monocytes (refer to Figure 5).** (A) RT‒qPCR analysis showing that LPS stimulation increased *cxcl10* expression in THP-1 cells, **P* < 0.05 compared with the Con group, n=3. (B) Representative Western blot showing that SiO_2_ has no effect on the expression of HSPA5 and DDIT3 in THP-1 cells. (C) Densitometric analyses of HSPA5 levels in five independent experiments; **P* < 0.05 vs. the 0 h group. (D) Densitometric analyses of DDIT3 levels in five independent experiments; **P* < 0.05 vs. the 0 h group.

**Supplementary Figure 4.**

**
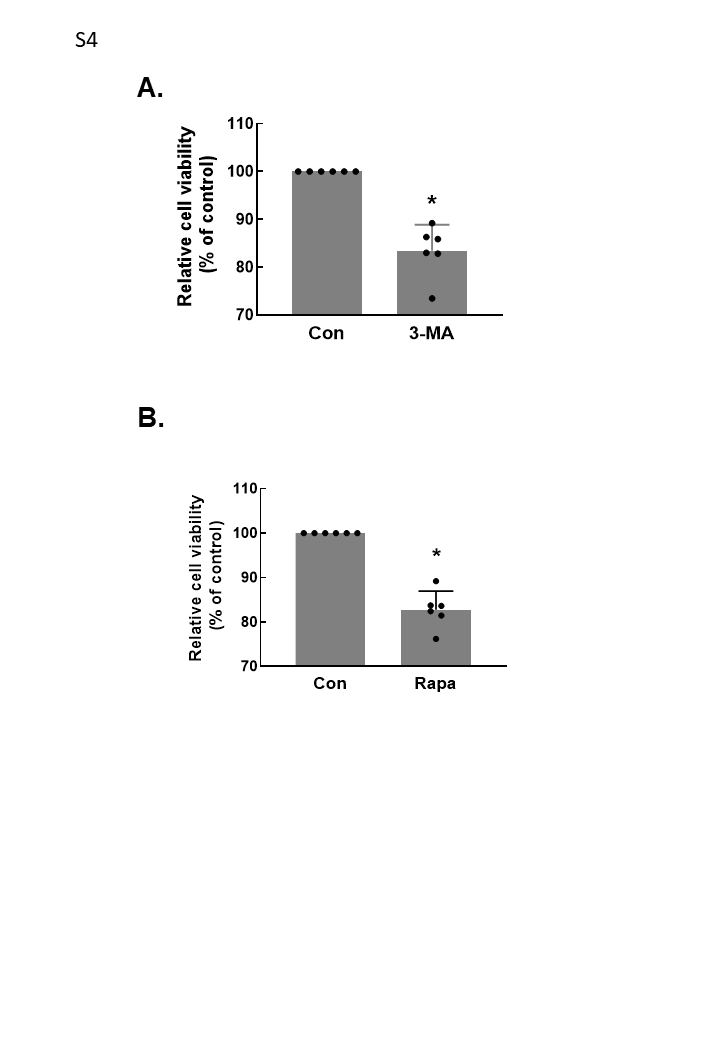
**

**Figure S4. The autophagy inhibitor 3-MA and the autophagy agonist rapamycin affect cell viability (refer to Figure 5).** (A) CCK-8 assay results showing that THP-1 cell viability was decreased by the autophagy inhibitor 3-MA; **P* < 0.05 vs. the Con group, n=5. (B) CCK-8 assay results showing that THP-1 cell viability was decreased by the autophagy agonist rapamycin; **P* < 0.05 vs. the Con group, n=5.

**Supplementary Figure 5.**

**
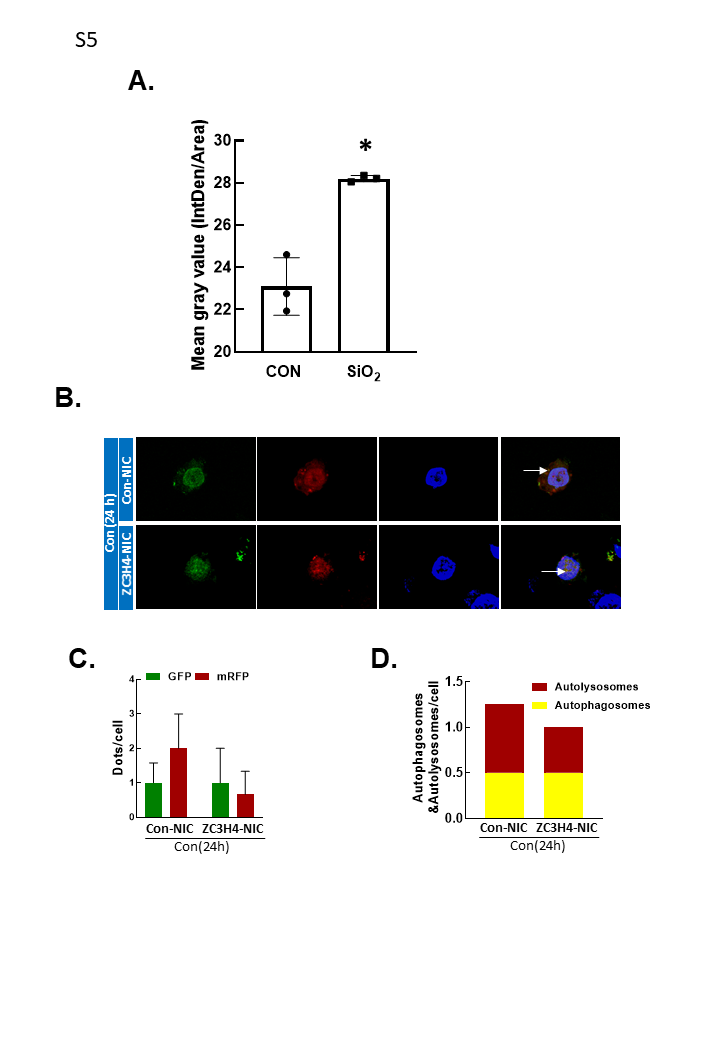
**

**Figure S5. ZC3H4 regulates IL-10 release through autophagic processes (refer to Figure 6 and Figure 7).** (A) Immunofluorescence staining quantification of ZC3H4 protein expression, **P* < 0.05 compared with the Con group. (B) Representative images of the fluorescence map showing that autophagy was not changed in THP-1 cells in the Con group and ZC3H4 protein knockdown. Scale bar, 80 μm. (C) Quantification of autophagy levels in THP-1 cells in the Con group after ZC3H4 protein knockdown. (D) Quantification of autolysosomes and autophagosomes in THP-1 cells in the Con group after ZC3H4 protein knockdown.
